# 

**Published:** 1865-05

**Authors:** 


### CONTENTS.

### ORIGINAL CONTRIBUTIONS.

ART. XVI. PUBLIC ADDRESS TO THE MEMBERS OF THE ILLINOIS STATE MEDICAL SOCIETY AND THE CITIZENS OF BLOOMINGTON, ON THE NATURE OF MEDICAL SCIENCE AND ITS RELATIONS TO THE COMMUNITY. Delivered May 2, 1865. By N. S. DAVIS, M.D., Chicago.............. 257

ART. XVII. ON DIURETICS IN THE TREATMENT OF MALARIOUS OR PERIODICAL FEVERS. Read to the Meeting of the Illinois State Medical Society, May 3, 1865. By N. WRIGHT, M.D., Chatham, Ill.,........... 269

### PROCEEDINGS OF SOCIETIES.

PROCEEDINGS OF THE ILLINOIS STATE MEDICAL SOCIETY FOR 1865,.......... 272

### SELECTIONS.

A Report on New Remedies. By Edward B. Stevens, M.D., Cincinnati. From the Transactions of the Ohio State Medical Society, 1864,........ 285

Lecture on the Results of the Surgical Treatment of Cancer. By T. Spencer Wells, F.R.C.S., Surgeon in ordinary to Her Majesty's Household,........ 293

The Epidemics in Russia and North Germany.* Report of the Medical Officer of the British Privy Council,........ 301

Small-Pox in Lahore,........ 303

Slitting the Cervix in Dysmenorrhœa,........ 304

Entozoa in Veal and Beef,........ 305

### BOOK NOTICES.

Hand-Book of Skin Diseases. For Students and Practitioners. By Thos. Hillier, M.D., London, Member of the Royal College of Physicians,... 306

A Vest-Pocket Medical Lexicon: Being a Dictionary of the Words, Terms, and Symbols of Medical Science. By D. B. St. John Rosa, M.D.,...... 307

Twenty-Second Annual Report of the Managers of the New York State Lunatic Asylum, for the year 1864,........ 307

A Radical Operation for Procidentia. By Thomas Addis Emmet, M.D., Surgeon to the State Woman's Hospital, New York,........ 307

London Lancet,........ 308

### EDITORIAL.

Illinois State Medical Society,........ 308

Annual Meeting of the American Medical Association,........ 308

Assassination of President Lincoln,........ 310

American Medical Association, Meeting of,........ 315

City Mortality.—Statistics for April,........ 316

French Medical Students,........ 317

Dr. Livingstone, contemplated visit to Africa,........ 317

An Ounce of Quinine at a Dose,........ 317

